# Cases Illustrative of the Use of Mercurial Ointment in Erysipelas, Swelled Leg, &c. &c.

**Published:** 1821-09

**Authors:** W. P. Little

**Affiliations:** the United States.


*Cases illustrative of the use of Mercurial Ointment in Erysipelas, Swelled Leg, &c. &c.* By W. P. Little, M.D. of the United States.

MUCH has been said of late of the efficacy of mercurial ointment in erysipelas, and the credit of introducing the practice seems very generally to be accorded to Dr. Dean, of Chambersburg. But the fact is, it originated with me, by whom it was communicated to that gentleman, when on a visit to this place, in February 1817; as can be fully proved.

I have hitherto been restrained from the assertion of my claims in this instance, by a wish to establish the utility of the practice by further experience; and, I can unaffectedly declare, no less by a reluctance to appear before the public in the character of a writer.

Case I.—In the winter of 1816, I was called to see a daughter of Captain Wilson, aged eight years, who had five weeks before accidentally run a thorn between the big and next toe of the right foot. The thorn was extracted, and no bad effects ensued till a few days before my visit, when the wound began to inflame and to be painful. Cataplasms of bread and milk were applied, by the direction of the mother, till the swelling subsided and the sore seemed healed. Two or three days had only elapsed, however, when she complained of great lassitude, and much pain in the head and neck, with nausea and occasional vomiting. An ounce of castor-oil was given by the mother, which, operating freely, afforded considerable relief. But, the next morning, the pain of the head and neck returned with increased vehemence, accompanied by a general soreness of the muscles of the body and extremities; and at noon she was seized with spasms, which were to an alarming degree, though of short duration. Early the following day, there was a recurrence of the spasms still more violent, and of longer continuance. I then was consulted in the case.

I found her with every symptom of confirmed tetanus, sometimes assuming the shape of opisthotonos, and at others of emphrosthotonos, and occasionally of trimus. After the operation of a cathartic, I opened and enlarged the wound which was healed, applied caustic to it, and subsequently an emollient poultice. The foot and ankle being swelled and erysipelatous in appearance, I also covered these parts with a blister. But, the disease continuing to advance, the inflammation extending up to the ilium, with appearances altogether unfavourable, though these and a variety of other remedies were tried, I resolved to bring about a salivation. To effect this purpose, I caused the strong mercurial ointment to be rubbed freely on the extremities, as well as on the throat, morning and evening, and in the interval had a saturated roller applied over the inflamed surfaces. As early as the third dressing, I perceived a manifest improvement in the swelling; and on the fourth day the local affection was entirely well, and with it a complete removal of the general disease.

Case II.—In 1820, I attended a female of sixty-five years of age, of a gross habit of body, labouring under a most extensive erysipelatous inflammation, which had located itself in the back. It originated from a small pimple, which appeared in the lumbar region, and, from the pain and hardness, was considered for many days as merely the beginning of a common boil. It continued to increase in size, and the hardness and redness to extend, with an intolerable itching and burning sensation; but it was not attended by any general derangement of the system, till three days prior to my visit, when I found her with a low fever, exceedingly debilitated, and with all the indications of gastric disturbance.

The inflammation had extended from ilium to ilium, and above the points of the scapulæ. It was of a dark red hue, very hard, and excessively painful to the touch, having the cuticle in different places raised, underneath which the aspect was dark and gangrenous.

The treatment was commenced with a dose of salts, and by covering the inflamed parts with strong mercurial ointment; directing it also to be rubbed in, morning and night.

The next day she was evidently better. Continuing nearly the same treatment, alternately purging and using nitrous powders, with the mercurial dressings, for five days longer, she was considered convalescent. But the part originally affected sloughed to the extent of three inches, leaving a large cavity over the spine, which gradually filled up. It is, however, worthy of remark, that, in consequence of improperly indulging in stimulating food, the edges of the wound became hard and erysipelatous, which affection was removed by the re-application of mercurial in place of basilicon ointment.

Case III.—Nearly about the same period, I attended a female child of Mr. Chesnut, aged four years, with one of the worst attacks of erysipelas which I ever witnessed. Commencing on the back of the neck, it soon pervaded the whole body. The face was much swelled, the eyes closed, the person generally œdematous, in many parts of a livid hue, with ichorous vesications. Her pulse was weak, small, and quick; the tongue furred, the breath fetid, and a heavy coma existed.

Entertaining the notion that the disease was of gastric origin, I began the management of the case with an emetic, which was followed by active purging with calomel and jalap; and in the intervals antimonial and cretaceous medicines were interposed.

The entire surface was dressed with strong mercurial ointment, though less was applied to the head than elsewhere.

The first dressing proved obviously beneficial, by relieving the burning and itching, which harassed, distressed, and rendered her restless and extremely fretful during the evening exacerbations. The inflammation rapidly declined after the second dressing, and in a few more applications of the ointment she was well.

Case IV.—On the 23d of January, 1817, Mrs. B., of a delicate constitution, was favourably delivered of her first child, and six days afterwards became ill of puerperal fever; from which, however, she soon recovered. On the 12th of February, I was again requested to see her, and found her labouring under that singular complaint called swelled leg,* attended by the usual symptoms of low fever.

Being exceedingly reduced by previous disease and copious depletion, I was precluded from the use of the lancet, and, after moderate evacuation of the bowels, I administered a combination of calomel and opium. Baffled in my attempts to cure the disease by this and many other measures, general and topical, without indeed producing even any very sensible alleviation, I determined to try the mercurial ointment, exactly as in the erysipelatous cases. To my great satisfaction, when I saw her four days afterwards, she was sitting up in bed, nursing her child, suffering no pain, the swelling nearly gone, and her condition in every way so much amended as no longer to require my attendance.

P.S.—Previous to the use of the ointment, I met with no disease more difficult to manage than erysipelas, in relation to topical remedies; but since, very little in this respect, governed, as I am, in the general treatment by the state of the system. I have found it beneficial in all the different species and grades of that disease, and, from parity of reasoning, would suppose it an appropriate remedy to confluent small-pox, and some cases of measles. Twice I have prescribed the ointment, mixed with charcoal, in gangrene,—once of the scrotum, and in another instance of the foot, with decisive advantage. It excited a healthy action, threw off the sloughs, established the suppurative process, and destroyed the fetor. In carbuncle, I have

---

* Phlegmatia dolens.

witnessed very beneficial effects from it, and also in chilblains and paronychia. Early applied in the latter affection, it often prevents suppuration. Last winter, I was consulted in a case of extensive inflammation of the ear, which had suppurated and discharged very freely for weeks; the pain attending it was most excruciating. After a time, the discharge ceased, and was followed by an extensive swelling at the back of the ear. Cataplasms of different kinds were applied for ten or twelve days, without reproducing the discharge or effecting any mitigation of pain: on the contrary, the swelling continued to increase in size and hardness. In this stage of the affection I was consulted, and, on examination, found the tumor to be scrofulous, attended with erysipelatous inflammation, which was becoming gangrenous. I directed the ointment to be freely applied to the swelling, and introduced into the ear. The good effects of the remedy were soon experienced, and, with the aid of such medicines as the case seemed to demand, an entire cure was readily accomplished.*

---

* In the last two years, (Dr. Chapman says,) the mercurial ointment has been a good deal employed in erysipelatous inflammation by Dr. Physick, and some other distinguished practitioners in this city; and, as far as we have been able to learn, with much success. There is a form of the disease, not noticed by our correspondent, in which we have seen it exceedingly beneficial. The case to which we allude makes its appearance very often in infants a few days after birth, and, in its more violent attacks, breaks out and diffuses itself over the lower portion of the belly, descending between the thighs to the breech, involving the genital organs.

It differs from common erysipelas in this, among other respects, that it seems mostly to be seated in the cellular membrane, which, becoming inflamed and thickened, produces a swelling hard and prominent. The appearance at first is florid, though not uniformly. Cases we have now and then met with, where there was a great degree of intumescence, without any discoloration, resembling very closely phlegmatia dolens. It is most apt to assume this character, we think, in adults or children some years old; and we have found it in every instance located in the thigh. Dressings of mercurial ointment, aided by copious evacuations of the primæ viæ, will generally effect relief in this disease, which hitherto has proved exceedingly intractable to other remedies.

As regards the utility of the ointment in the swelled leg of puerperal women, our own experience does not warrant us to express an opinion. We learn, however, from Dr. Dewees, whose opportunities of determining the point have been ample, that he does not repose much confidence in it. Thirty years ago, he tells us, it was used by his preceptor, Dr. Smith, and probably by other physicians of that period. He has also in his own practice tried it repeatedly.

We have long known a solution of corrosive sublimate as a popular remedy in inflammations of this nature, from poisons; and, in the proportion of one grain to an ounce of water, has been strongly recommended in genuine erysipelas by Dr. Schott, an excellent practitioner of this place. Citron ointment we have for many years directed in one variety of erysipelas, that of the nose, symptomatic of disordered chylopoietic viscera; and a drachm or two of calomel to the ounce of lard, or equal portions of calomel and chalk dusted on the part, afford the most effectual means with which we are conversant in the erysipelatous eruptions incident to dentition.—(*Philadelphia Journal of the Medical and Physical Sciences.*)

